# Simultaneous dose distribution and fluence prediction for nasopharyngeal carcinoma IMRT

**DOI:** 10.1186/s13014-023-02287-4

**Published:** 2023-07-04

**Authors:** Yongbao Li, Wenwen Cai, Fan Xiao, Xuanru Zhou, Jiajun Cai, Linghong Zhou, Wen Dou, Ting Song

**Affiliations:** 1 Department of Radiation Oncology, State Key Laboratory of Oncology in South China, Collaborative Innovation Center for Cancer Medicine, Guangdong Key Laboratory of Nasopharyngeal Carcinoma Diagnosis and Therapy, Sun Yat-sen University Cancer Center, Guangzhou, 510060 China; 2 School of Biomedical Engineering, Southern Medical University, Guangzhou, 510515 China; 3 Zhujiang Hospital, Southern Medical University, Guangzhou, 510282 China

**Keywords:** Dose distribution prediction, Fluence prediction, Nasopharyngeal carcinoma, IMRT, Shared encoder network

## Abstract

**Background:**

Current intensity-modulated radiation therapy (IMRT) treatment planning is still a manual and time/resource consuming task, knowledge-based planning methods with appropriate predictions have been shown to enhance the plan quality consistency and improve planning efficiency. This study aims to develop a novel prediction framework to simultaneously predict dose distribution and fluence for nasopharyngeal carcinoma treated with IMRT, the predicted dose information and fluence can be used as the dose objectives and initial solution for an automatic IMRT plan optimization scheme, respectively.

**Methods:**

We proposed a shared encoder network to simultaneously generate dose distribution and fluence maps. The same inputs (three-dimensional contours and CT images) were used for both dose distribution and fluence prediction. The model was trained with datasets of 340 nasopharyngeal carcinoma patients (260 cases for training, 40 cases for validation, 40 cases for testing) treated with nine-beam IMRT. The predicted fluence was then imported back to treatment planning system to generate the final deliverable plan. Predicted fluence accuracy was quantitatively evaluated within projected planning target volumes in beams-eye-view with 5 mm margin. The comparison between predicted doses, predicted fluence generated doses and ground truth doses were also conducted inside patient body.

**Results:**

The proposed network successfully predicted similar dose distribution and fluence maps compared with ground truth. The quantitative evaluation showed that the pixel-based mean absolute error between predicted fluence and ground truth fluence was 0.53% ± 0.13%. The structural similarity index also showed high fluence similarity with values of 0.96 ± 0.02. Meanwhile, the difference in the clinical dose indices for most structures between predicted dose, predicted fluence generated dose and ground truth dose were less than 1 Gy. As a comparison, the predicted dose achieved better target dose coverage and dose hot spot than predicted fluence generated dose compared with ground truth dose.

**Conclusion:**

We proposed an approach to predict 3D dose distribution and fluence maps simultaneously for nasopharyngeal carcinoma patients. Hence, the proposed method can be potentially integrated in a fast automatic plan generation scheme by using predicted dose as dose objectives and predicted fluence as a warm start.

## Background

Radiation therapy is one of the important means to treat patients with cancer. With the aim to give sufficient high dose coverage to target and minimize the dose to nearby normal tissues and organs, the intensity-modulated radiation therapy (IMRT) technique is most commonly used in clinics, which modulates the high-energy photon beam intensity by external devices such as multi-leaf collimators (MLCs) [1, 2]. In addition, IMRT has the advantage to deliver conformal doses to targets and protect adjacent normal tissues and organs, therefore, it is widely used for head and neck cancer (nasopharyngeal carcinoma, etc.) treatment with complex anatomical structures [3, 4]. At present, treatment planning for IMRT is typically completed in a treatment planning system (TPS) via inverse plan optimization [5], where manual and tedious dose objectives and constraints tuning procedure is included [6]. Consequently, the planning process is time/resource-consuming and the plan quality largely depends on the experience of the planners [7].

Thus, knowledge-based planning (KBP) methods were introduced to automate the planning process to enhance the plan quality and consistency and improve planning efficiency for IMRT [8–13]. Initially, it was done by dose objectives prediction to guide the subsequent inverse optimization or so-called dose mimicking [14]. Dose objective prediction aims to build a relationship between the anatomical structures and dosimetric characteristics of patients based on machine learning from a large number of prior plans. Meanwhile, previous studies focused on specific dose criteria or dose volume histograms (DVHs) prediction via traditional machine learning methods [15–18], the latest development predicted the patient 3D dose distribution by using deep convolutional neural networks (CNNs), particularly U-Net and its derivatives [19–22]. The later dose mimicking step gets a plan to restore these predicted dose objectives by inverse optimization.

Moreover, recent studies had moved KBP methods to another stage, namely predicting deliverable fluence directly bypassing the inverse optimization, the final plan would be obtained by MLC leaf sequencing of the predicted fluence. Among these studies, Lee et al. used a 2D U-Net to generate fluence maps from organ contours and field doses viewed from the beam’s eye view for prostate IMRT plans [23]. Meanwhile, Wang et al. further used a CNN to initially predict field doses, and then converted these 3D field doses to 2D field doses and input them to another CNN to predict fluence maps for pancreas IMRT plans [24, 25]. Furthermore, Ma et al. utilized the idea of inverse mapping from the projections of the desired plan dose to generate fluence maps for volumetric modulated arc therapy plans [26]. Subsequently, Li et al. developed a deep learning algorithm to predict fluence from two kinds of 2D projection maps representing the patient’s anatomical information for prostate IMRT [27]. Then, they later extended the study to predict fluence for head-and-neck (only low-risk target was included) IMRT plans [28]. Yuan et al. trained a two-stage CNN to predict fluence for cervical cancer IMRT plans [29].

Although success has been achieved for fluence prediction, particularly for IMRT plans, there are still some other issues that need to be addressed. First, delivering the plan generated by directly leaf sequencing of the solely predicted fluence is risky in a clinic, as it is difficult to know whether or not the predicted fluence is an optimal solution for a patient and the predicted fluence is realistic enough to deliver without significant plan quality loss. Predicting 2D fluence map directly from 3D structure set has a larger uncertainty than predicting 3D dose since of 3D-to-2D dimension issue and weak inherent data correlation. Even though patient achievable 3D plan dose distribution is known, an inverse optimization problem is usually solved to get the corresponding 2D fluence map because of problem degradation [26]. In addition, converting 2D fluence map to a final deliverable plan includes a MLC leaf motion calculation and accurate dose calculation step, plan quality loss is typically happened after conversion especially when the predicted fluence is not realistic enough. On the contrary, 3D dose distribution prediction has been well studied by many researchers and proved to be feasible for guiding automatic plan optimization [20, 30, 31]. Hence, patient-specific dose information prediction is still desired to guide further optimization of plan generated from fluence prediction. Once the predicted fluence is known as not optimal, further optimization can be continued by using predicted patient-specific dose information as objectives and predicted fluence as initial values. Second, the above studies need either field dose predictions as model inputs or pre-determined 2D feature map extraction steps for fluence prediction [23–29]. Considering dose and fluence are highly related, dose distribution prediction and fluence prediction can be unified under the same framework. Previously existing 3D dose distribution prediction networks were then utilized without additional inputs and complex network architectures for fluence prediction. Third, most previous studies focused on fluence predictions on relatively simple tumor sites (such as abdomen and pelvis), more complicated tumor sites with complex geometric relationship between targets and normal organs have not been fully investigated.

Therefore, in this work, we proposed an approach to simultaneously predict 3D dose distribution and fluence maps for more complicated nasopharyngeal carcinoma patients (five targets and seventeen organs included) treated with nine-beam IMRT. A shared encoder network extended from 3D U-Net was proposed, 260 patients were used to train an optimal model, and both dose distribution prediction and fluence prediction results were presented and evaluated with an independent test set of 40 patients. Herein, the developed method can provide additional dose information to guide further plan optimization for automatic plan generation based on fluence prediction.

## Methods

### Patient data and processing

The datasets were collected from 340 nasopharyngeal carcinoma patients treated with IMRT at Sun Yat-sen University Cancer Center. The Ethics Committee of Sun Yat-sen University Cancer Center approved the use of patient treatment plan samples in this study. All patients were irradiated with nine equally spaced beams (0°, 40°, 80°, 120°, 160°, 200°, 240°, 280°, and 320°) and 6 MV photon beam energy by the same treatment machine of Varian Trilogy system (Varian Medical Systems, Palo Alto, CA, USA). The datasets were randomly separated into training, validation, and test sets with 260, 40, and the remaining 40 cases, respectively.

Then, we extracted CT images, contours of planning target volumes (PTVs) and organ at risks (OARs), dose distribution, and nine-beam fluence maps from DICOM files of each patient. Herein, the PTV contours were expressed as a 3D mask filled with prescription doses. Five PTVs named ‘PTV-GTV’, ‘PTV-1’, ‘PTV-2’, ‘PTV-LN(L)’ (PTV of left lymphonodus), and ‘PTV-LN(R)’ (PTV of right lymphonodus) were considered, the maximum prescription dose of PTVs where the voxel belonged was assigned to each voxel of the mask. The prescription doses of PTV-GTV/PTV-1/PTV-2 had two combinations of 7000 cGy/6400 cGy/5800 cGy and 7000 cGy/6000 cGy/5400 cGy. In addition, there were six prescription dose levels for PTV-LN(L) and PTV-LN(R), the possible values were 6000 cGy, 6200 cGy, 6400 cGy, 6600 cGy, 6800 cGy, and 7000 cGy. Moreover, seventeen OARs were considered, including body, brainstem, spinal cord, chiasm, tongue, left and right optic nerves, left and right lens, left and right temporal lobes, left and right mandibles, left and right temporomandibular joints, and left and right parotid glands. Each OAR was represented by a binary mask with one assigned inside the OAR and zero outside the OAR. All CT images, PTV and OAR masks, as well as dose volumes, were interpolated with a resolution of 2.5 mm × 2.5 mm × 2.5 mm and centered at a plan isocenter with a transverse slice size of 224 × 224.

The fluence maps were calculated based on the beam control point sequences by weighted summation of intermediate MLC and jaw transmission masks for each field. The dosimetric leaf gap and MLC transmission values were obtained from the Varian Eclipse treatment planning system. Furthermore, the fluence maps of nine beams were calculated with a resolution of 2.5 mm × 2.5 mm and then concatenated to a 3D matric with a size of 9 × 160 × 160. Consequently, the data of each case contained one CT volume, one PTV mask, seventeen OAR masks, one dose volume, and one fluence volume.

### Dose and fluence prediction network

**Fig. 1:**
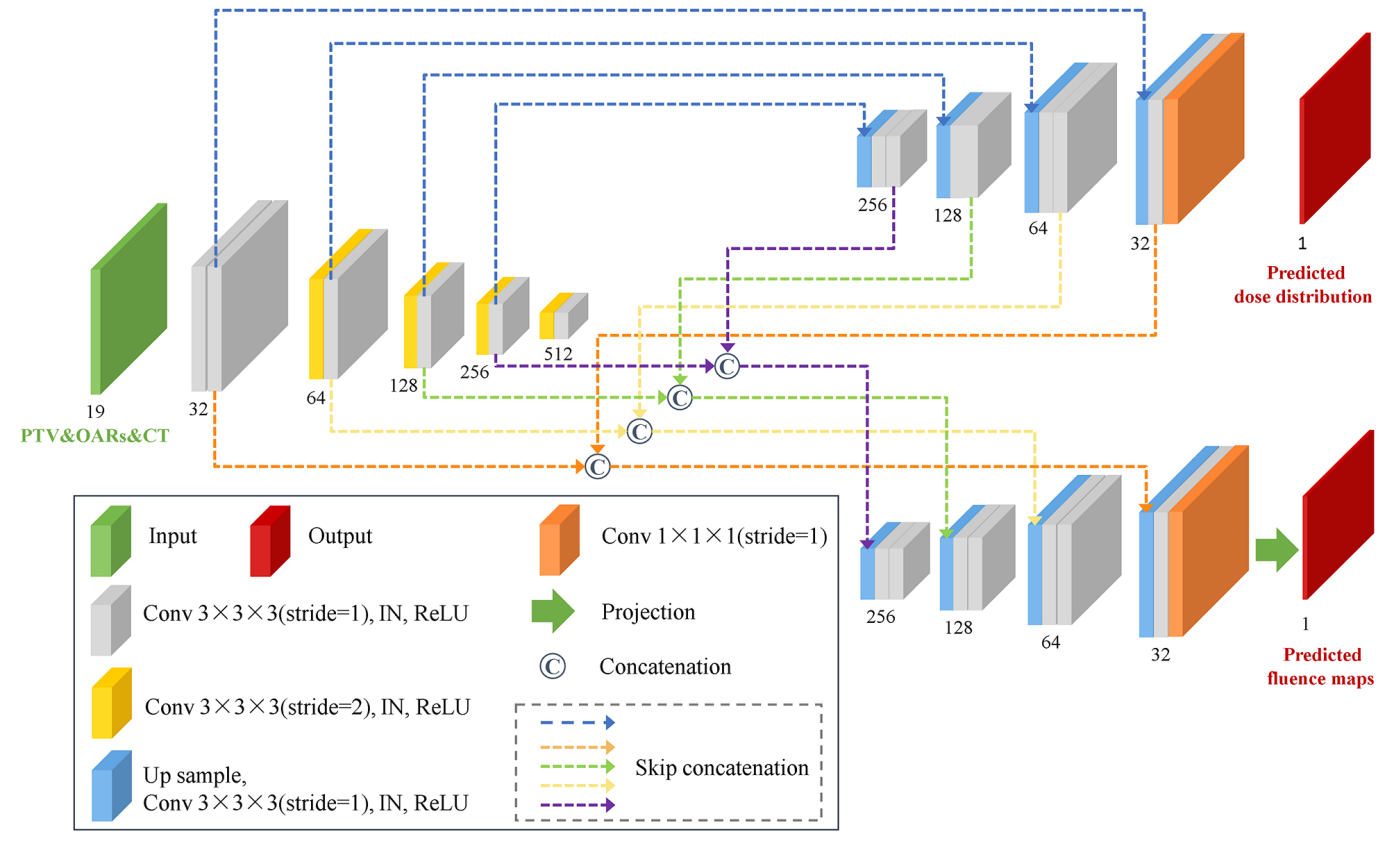
The architecture of the shared encoder network

Enlightened by the U-Net network with an end-to-end encoder-decoder structure which was widely used for patient 3D dose prediction tasks [21, 32, 33], we proposed a shared encoder network with minimal adjustments to the previous 3D U-Net to simultaneously generate dose distribution and fluence maps (Fig. 1). The same inputs were used for both dose distribution and fluence prediction, neither field dose predictions nor feature map pre-calculations were needed.

The network architecture included one encoding path with five resolution levels and two decoding paths with four resolution levels. Herein, one PTV mask, seventeen OAR masks, and one CT image were used as independent channels for network input, hence, there were nineteen channels in total. In the encoding path, conventional convolution and down sampling operations were employed to extract the key features and reduce the resolution of images. The first level consisted of two convolution operations with a kernel size of 3 × 3 × 3 and stride of 1. Meanwhile, for the below four levels of encoding path, each included one 3 × 3 × 3 convolution with a stride of 2 for down sampling and one 3 × 3 × 3 convolution with a stride of 1 to learn features. After each down sampling, the channel of the feature maps was doubled and the size was halved. Consequently, the channel of feature maps was increased from 32 to 512 and the size was contracted from 32 × 224 × 224 (slice × height × width) to 2 × 14 × 14.

In addition, in two decoding paths, we used up samplings, convolutions, and skip concatenations to restore image details and sizes. The trilinear interpolations and convolutions were employed in up sampling. Each level had two 3 × 3 × 3 convolutions with a stride of 1 except the last layer which only had one 3 × 3 × 3 convolution and one 1 × 1 × 1 convolution. Skip concatenations to connect corresponding feature maps from the encoding path to the decoding path were utilized to recover the lost information during down samplings [34]. In addition to connecting the encoder, the decoding path of fluence generation also connected the decoding path of dose generation to propagate the learned dose features at different resolution levels. The instance normalization (IN) and rectified linear unit (ReLU) activation were followed with each 3 × 3 × 3 convolution to prevent over-fitting and gradient explosion.

The output of the dose generation decoding path was 1 channel and the size was recovered to 32 × 224 × 224. However, for the output of fluence generation decoding path, there was a dimension issue between 3D volume feature space to 2D fluence space. Considering the issue, we introduced a 3D to 2D geometric projection operation to obtain the fluence map, the projection was conducted by a matrix and vector multiplying operation as $f = {P^T} \cdot v$, where $f$ was the fluence map, $v$ was the feature volume which predicted by the network, $T$ was the transpose operation, and $P$ was the geometric projection matrix with its element *P*_*ij*_, thereby indicating that the *i*-th voxel receives fluence contribution from the *j*-th beamlet with unity intensity. *P*_*ij*_ was calculated by only considering the inverse square effect and ${P}^{T}$ was stored as a sparse matrix. Hence, the above geometric projection operation can be easily inserted into the forward and backward propagation process of the network training. The loss function between predictions (including dose distribution and fluence map) and ground truth was calculated to update the network parameters.

### Training and evaluation

#### Network training

We randomly chose continuous 32 slices with at least one slice containing the nonzero value of the PTV mask as the input data patch because the number of slices for each patient is different and the memory of GPU is limited. Consequently, one PTV mask patch, seventeen OAR mask patches, and one CT image patch were stacked to 4D matric with a final size of 19 × 32 × 224 × 224. As labeling data, dose distribution patch (32 × 224 × 224) and fluence map patch (9 × 32 × 160) at the corresponding position were also extracted. Before training, the PTV mask and dose distribution were normalized by 7000 cGy, the fluence maps were normalized by 2000 monitor units (MUs), and the values of the CT image were first trimmed to the range of -1024–2000 HU and then were normalized by 2000 HU. During training, the input data of the network was augmented to expand the datasets and avoid over-fitting. Herein, two data augmentation ways were used: the whole 32 slices were randomly flipped in the left-right direction with a probability of 0.6 and randomly rotated around the superior-inferior axis at one of the degrees at {40°, 80°, 120°, 160°, 200°, 240°, 280°, and 320°} with a probability of 0.4, which meant there was a probability of (1-0.6) × (1-0.4) = 0.24 that augmentation was not used for a case in a training epoch. The dose distribution and fluence maps of the nine beams were also transformed based on the augmentation way of input data. The validation set was not augmented and only slices that contained a nonzero value of the PTV mask were used to choose the model achieved the best high dose region prediction accuracy.

Moreover, both dose and fluence maps used mean square error (MAE) between predicted values and ground truth values as a loss function. Dose MAE was calculated inside the patient body, and fluence MAE was calculated inside the projected PTVs in beams-eye-view with 5 mm margin. As to be noted, the edges of fluence map patches were not fully covered by the corresponding patient volume patches with the same slices because of the divergence of the radiation beam, hence, the predicted fluence patch at the edge region was not accurate after the truncated geometric projection. As such, we only calculated fluence loss with the middle part of the fluence map patch with a size of 9 × 26 × 160. After the model was trained, we used a sliding window fashion with some overlap regions to get the full fluence map as detailed in the Evaluation section. Finally, we took the sum of dose loss and fluence loss as the total loss function.

For the setting of hyperparameters, Adam [35] was used as an optimizer to minimize the loss function and batch size was set to 2. The initial learning rate was set to 0.0003, and the ReduceLROnPlateau scheduler was employed to reduce the learning rate by 30% when the validation loss did not improve after training 4 epochs. The network training was completed with Python 3.8 and Pytorch 1.10.1 on an NVIDIA RTX Titan GPU with 24 GB memory. Meanwhile, for the training of the shared encoder network, a total of 150 epochs were used and there were 400 iterations in each epoch.

### Evaluation

A total of 40 independent patient plans were used for testing to demonstrate the feasibility of our proposed network in simultaneous dose distribution and fluence maps prediction task. Thus, we utilized the sliding window method to sample the input data of each patient into several patches with a slice stride size of 24 (8 overlap slices) to generate the whole 3D dose distribution and fluence maps and then feed them into the trained network. The corresponding dose and fluence patches were then generated and collectively combined using a logarithmic function to smooth the overlap regions. The predicted fluence maps were then imported back to Eclipse treatment planning system (version 15.6) for MLC leaf motion calculations (Varian LMC 15.6.03) and final dose calculations (AAA 15.6.03), and then normalized to have the same PTV-GTV prescription dose coverage as original plan. Fluence map difference, MAE (%), structural similarity index (SSIM) [36], and global gamma passing rates with a threshold of 0% and 10% at the criteria of 3%/3mm were used to evaluate the predicted fluence maps accuracy. The comparison between predicted doses, predicted fluence generated doses and ground truth doses was also conducted by using dose distribution differences, DVH curves, and clinical indices which including the 95% volume received dose (D_95%_), mean dose (D_mean_) and max dose (D_max_) of the structure. Statistical differences were evaluated by Wilcoxon signed-rank tests at a 0.05 significance level.

## Results

As for model training, it took 6 days to train the proposed shared encoder network. After the model was trained, it took 18 s to generate both doses and fluences for one patient.

### Fluence evaluation

**Fig. 2:**
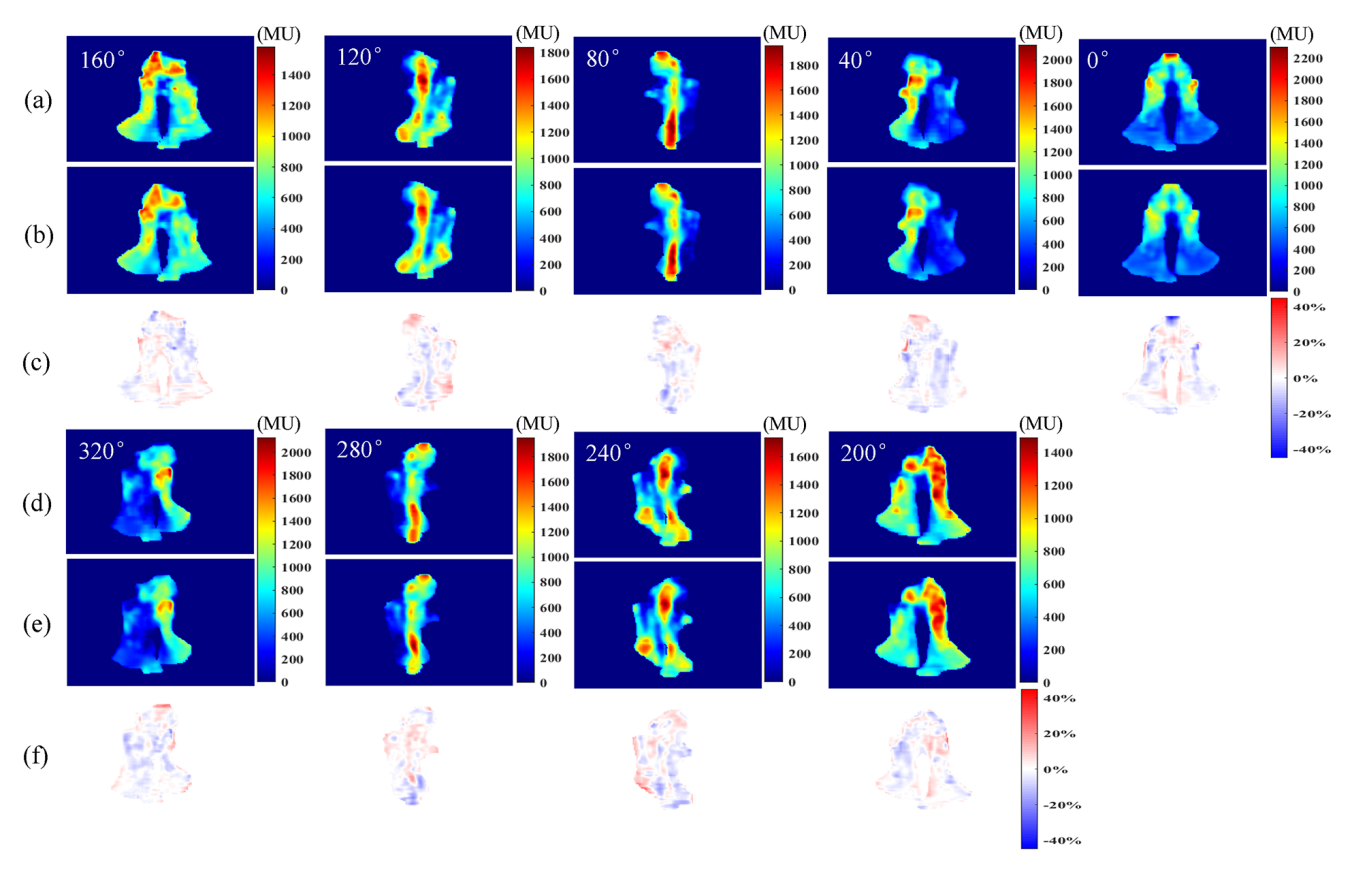
The fluence maps comparison at all nine beam angles for a test case. (**a**)(**d**) are the fluence maps of ground truth, (**b**)(**e**) are the predicted fluence by shared encoder network, (**c**)(**f**) are the fluence difference map between ground truth and prediction normalized by 2000 HU

Figure 2 presents the comparison of fluence maps at nine beam angles for a test case. The pixel-level difference maps were achieved through subtraction between predicted fluence and ground truth fluence and then normalized with 2000 HU which closed to the maximum of most ground truth fluence maps. The predictions show similar fluence modulation and morphological features as ground truth, the high-intensity region to irradiate tumors and the low-intensity region to spare OARs were both recovered in the corresponding place. Yet, the visual differences can be also observed particularly in high-intensity regions.

**Fig. 3:**
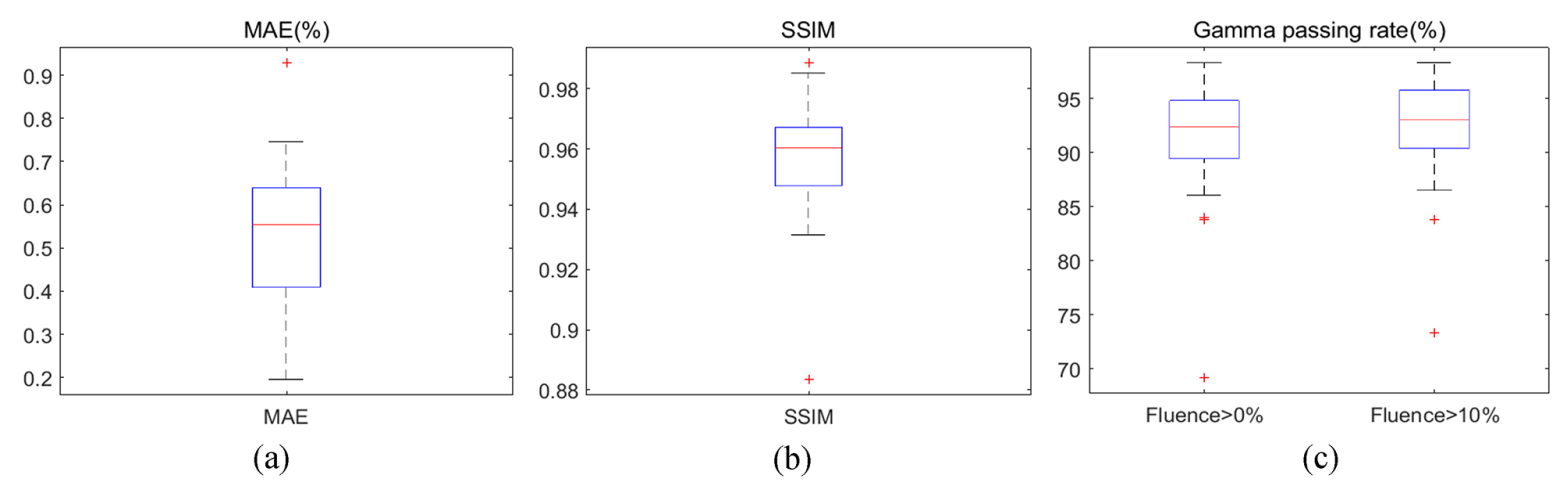
Quantitative comparison results between ground truth fluence and predicted fluence with (**a**) MAE, (**b**) SSIM, (**c**) global gamma passing rate with 0% threshold and 10% threshold at 3%3mm criteria, respectively

Meanwhile, Fig. 3 lists the average gamma passing rate, MAE, and SSIM for 40 test patients. The average global gamma passing rate with criteria of 3%/3mm and thresholds of 0% and 10% were both large than 92%. The MAEs were normalized by the maximum value of ground truth and showed average values of 0.53% ± 0.13%, which was evaluated within the projected PTVs in beams-eye-view with 5 mm margin. In addition, the SSIM also showed higher values of 0.96 ± 0.02 and further quantified the structural similarity of the predicted fluence maps with ground truth.

### Dose evaluation

**Fig. 4:**
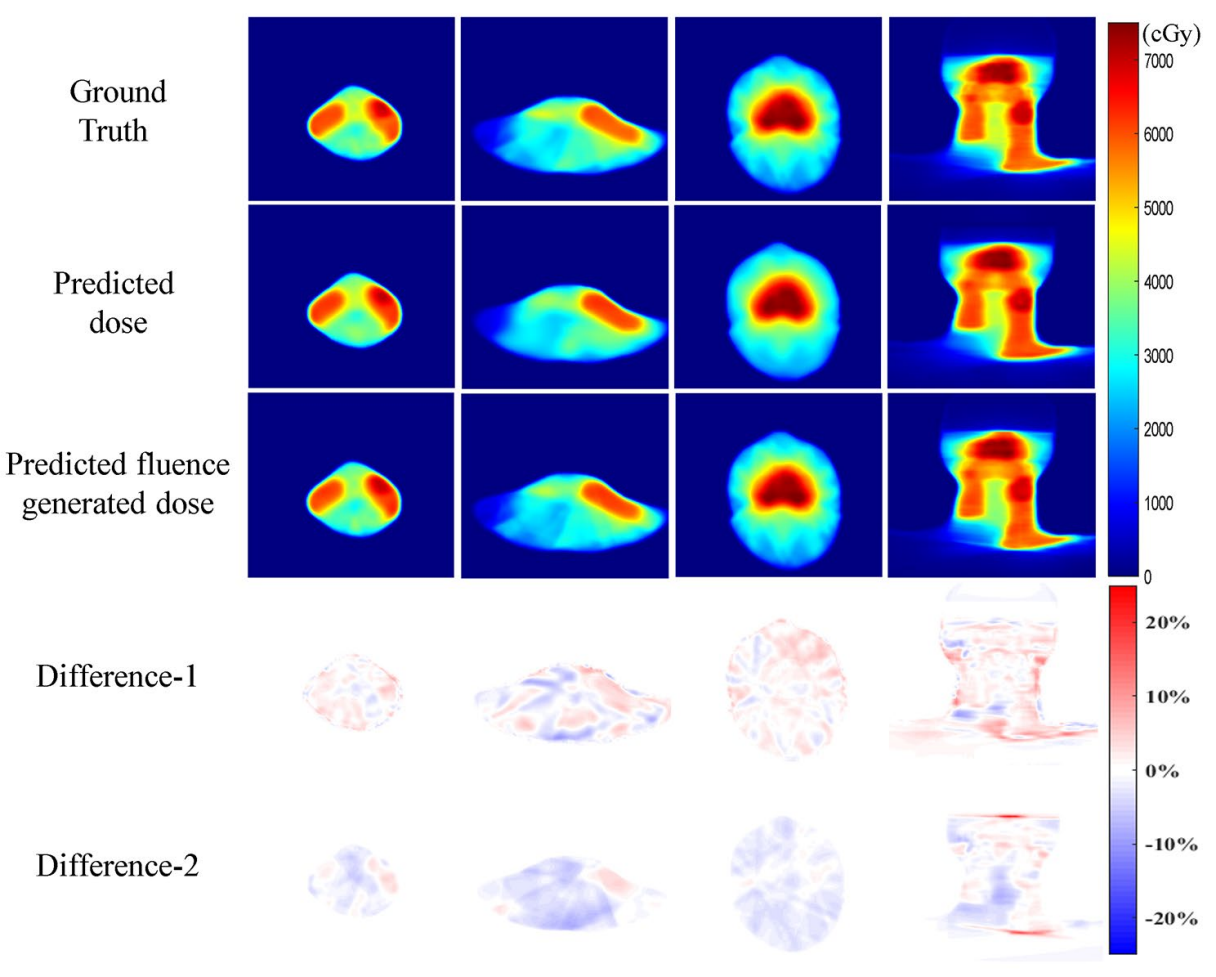
The dose distribution comparison for a test case. The first three columns are transverse sections and the fourth column is coronal section. Difference-1: the difference between ground truth dose and predicted dose by shared encoder network. Difference-2: the difference between ground truth dose and predicted fluence generated dose normalized by the prescription dose of 7000 cGy

**Fig. 5:**
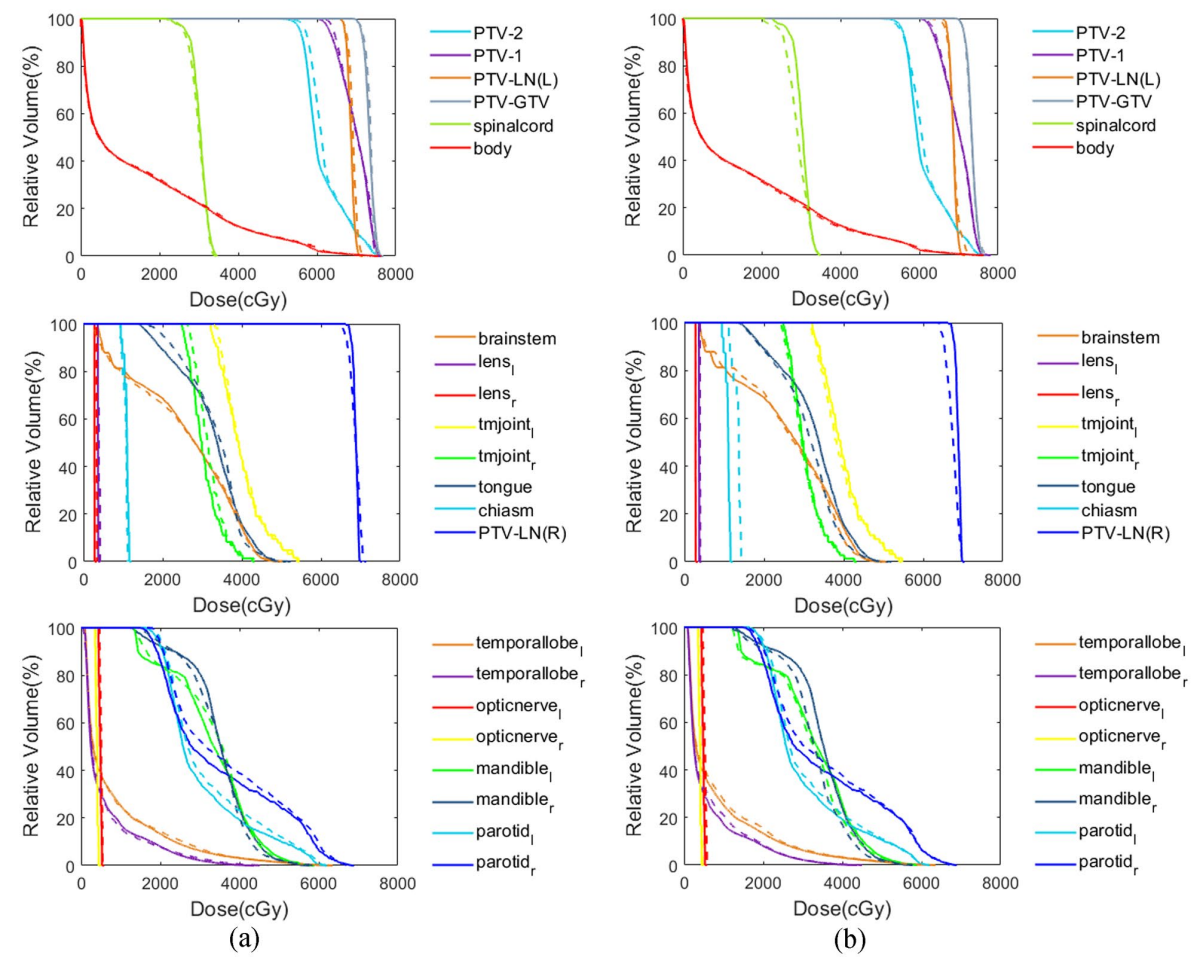
(**a**) The DVHs comparison between ground truth dose (solid line) and predicted dose by shared encoder network (dashed line). (**b**) The DVHs comparison between ground truth dose (solid line) and predicted fluence generated dose (dashed line)

Figure 4 shows the comparison of dose distributions and differences in three transverse sections and one coronal section. The difference maps were obtained by subtracting between predicted doses, predicted fluence generated doses and ground truth doses voxel by voxel and then normalized by the prescription dose of 7000 cGy. Both predicted doses and predicted fluence generated doses show high similarity with ground truth doses. The comparisons of DVH curves for five PTVs and seventeen OARs from the same patient in Fig. 4 were illustrated in Fig. 5. The solid lines and dashed lines represent the DVH curves of ground truth and predictions respectively. Most DVHs from the model predicted doses and model predicted fluence generated doses show minor differences with ground truth (D_mean_ difference less than 1% of prescription dose), whereas left and right parotids show a relatively large difference (D_mean_ difference large than 2% of prescription dose), which may be due to a complex dose pattern given that parotids were overlapped with high dose PTV-LNs and PTV-2. In addition, PTV-LN(R) and chiasm from predicted fluence generated dose show significantly under-dose and over-dose than ground truth, while predicted dose only has a minor difference with ground truth.

The clinical indices were listed in Table 1 to assess the dose coverage of PTVs and the exposure dose of OARs. The average differences between predicted dose, predicted fluence generated dose and ground truth were less than 1 Gy for most structures, meanwhile, optic nerves, chiasm, and lens show a large dose difference with ground truth because of small organ sizes and inadequate voxel sampling.

**Table 1:** The clinical indices comparison for forty test patients with the unit of Gy (mean ± standard deviation). p1: significant difference between ground truth and predicted dose by shared encoder network. p2: significant difference between ground truth and predicted fluence generated dose. Results with P < 0.05 indicated statistical significance and were labeled with *

Structures	Clinical indices (Gy)	Ground truth	Predicted dose	Predicted fluence generated dose	p1	p2
PTV-GTV	D_95%_	71.1 ± 0.6	71.2 ± 0.4	70.5 ± 1.1	0.24	0.00^*^
	D_mean_	73.4 ± 0.6	73.1 ± 0.4	73.6 ± 0.5	0.06	0.00^*^
	D_max_	76.8 ± 0.8	76.1 ± 0.8	78.0 ± 1.3	0.00^*^	0.00^*^
PTV-1	D_95%_	63.8 ± 1.8	64.5 ± 1.6	63.9 ± 1.5	0.00^*^	0.13
	D_mean_	70.2 ± 1.1	70.1 ± 1.1	70.4 ± 1.1	0.38	0.00^*^
	D_max_	76.8 ± 0.9	76.1 ± 0.8	78.0 ± 1.3	0.00^*^	0.00^*^
PTV-2	D_95%_	57.1 ± 1.7	57.6 ± 1.4	56.3 ± 1.6	0.00^*^	0.00^*^
	D_mean_	63.7 ± 2.0	63.6 ± 1.7	63.6 ± 1.9	0.62	0.66
	D_max_	76.7 ± 1.2	75.9 ± 1.5	77.8 ± 1.7	0.00^*^	0.00^*^
PTV LN(L)	D_95%_	67.6 ± 2.5	66.6 ± 2.2	66.4 ± 2.4	0.00^*^	0.00^*^
	D_mean_	68.9 ± 2.6	68.7 ± 2.2	68.7 ± 2.5	0.40	0.78
	D_max_	70.9 ± 3.0	71.4 ± 2.7	71.9 ± 3.2	0.02^*^	0.00^*^
PTV LN(R)	D_95%_	67.3 ± 2.8	66.5 ± 2.1	66.1 ± 2.6	0.00^*^	0.00^*^
	D_mean_	68.6 ± 2.9	68.4 ± 2.3	68.2 ± 2.7	0.45	0.17
	D_max_	70.3 ± 3.3	70.9 ± 3.0	71.1 ± 3.3	0.01^*^	0.02^*^
Spinal cord	D_max_	35.5 ± 1.3	36.0 ± 1.6	35.9 ± 2.1	0.02^*^	0.16
Brainstem	D_max_	55.9 ± 5.5	55.1 ± 4.9	55.5 ± 4.9	0.03^*^	0.17
Left optic nerve	D_max_	35.8 ± 21.4	37.5 ± 22.4	37.0 ± 22.3	0.01^*^	0.24
Right optic nerve	D_max_	36.5 ± 21.4	38.0 ± 22.4	37.4 ± 21.7	0.03^*^	0.09
Chiasm	D_max_	43.8 ± 19.6	46.5 ± 19.2	45.5 ± 20.4	0.00^*^	0.05
Left len	D_max_	6.3 ± 2.9	6.7 ± 3.0	6.0 ± 2.6	0.24	0.40
Right len	D_max_	6.4 ± 3.3	6.8 ± 3.5	6.2 ± 3.2	0.28	0.95
Left temporal lobe	D_mean_	18.9 ± 6.9	19.4 ± 6.7	18.7 ± 6.7	0.03^*^	0.32
Right temporal lobe	D_mean_	19.5 ± 7.5	19.4 ± 6.6	18.7 ± 6.6	0.51	0.00^*^
Left mandible	D_mean_	43.1 ± 6.2	42.7 ± 5.7	42.3 ± 6.0	0.02^*^	0.00^*^
Right mandible	D_mean_	42.2 ± 4.7	42.1 ± 4.2	41.6 ± 4.7	0.93	0.00^*^
Left parotid gland	D_mean_	38.4 ± 4.4	38.7 ± 4.8	38.5 ± 5.0	0.29	0.96
Right parotid gland	D_mean_	38.5 ± 3.9	39.0 ± 4.0	38.6 ± 4.4	0.08	0.96
Body	D_mean_	19.0 ± 4.0	20.2 ± 2.9	18.7 ± 4.0	0.01^*^	0.00^*^
Tongue	D_mean_	43.0 ± 4.3	42.8 ± 3.9	42.5 ± 4.4	0.67	0.02^*^
Left temporo-mandibular joint	D_mean_	43.6 ± 8.7	43.7 ± 8.1	43.3 ± 8.7	0.97	0.05
Right temporo-mandibular joint	D_mean_	42.9 ± 9.5	42.9 ± 8.9	42.2 ± 9.5	0.91	0.00^*^

**Fig. 6:**
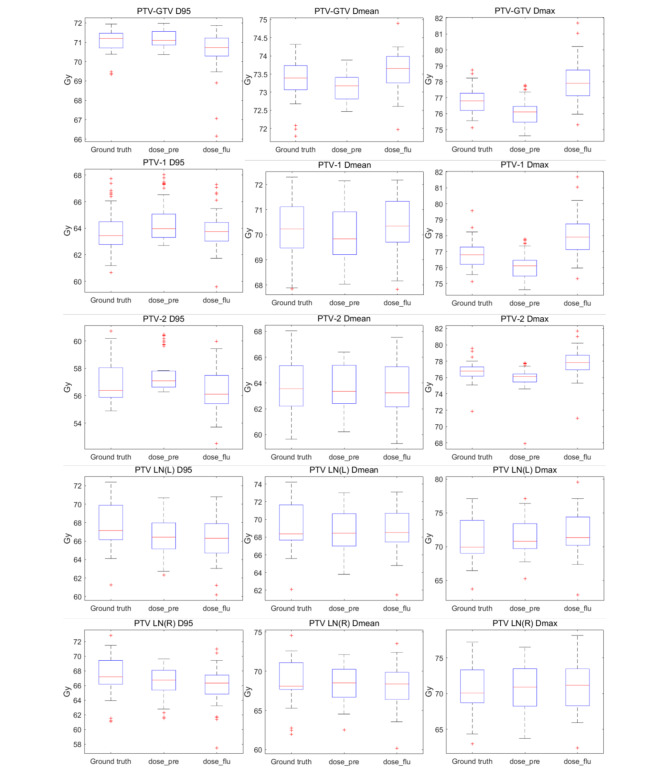
Box plot comparisons of dosimetric results between ground truth doses, predicted doses (dose_pre) and predicted fluence generated doses (dose_flu) for five targets

**Fig. 7:**
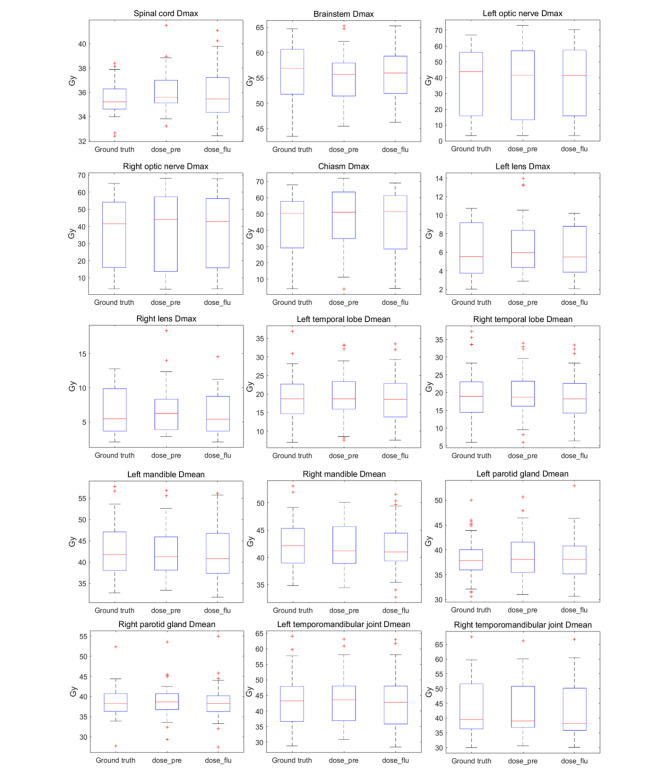
Box plot comparisons of dosimetric results between ground truth doses, predicted doses (dose_pre) and predicted fluence generated doses (dose_flu) for fifteen OARs

Figure 6 and Fig. 7 further show the box plot comparisons of dosimetric results between ground truth doses, predicted doses and predicted fluence generated doses for five targets and most OARs. Overall, the predicted fluence generated dose show a relatively lower target dose coverage and higher dose hot spot than ground truth and predicted dose. For OARs, no significant differences were found between predicted dose and predicted fluence generated dose.

## Discussion

In this study, we proposed a shared encoder network for simultaneous 3D dose distribution and fluence maps prediction for nasopharyngeal carcinoma patients. The proposed network successfully predicted similar dose distribution and fluence maps compared with ground truth. The predicted fluence maps were further imported back into treatment planning system and allowed to generate a dose distribution close to ground truth. In addition, patient-specific dose information prediction can be used to guide further optimization of plan generated from fluence prediction. Once the predicted fluence is known as not optimal, further optimization can be continued by using predicted patient-specific dose information as objectives and predicted fluence as initial values.

Studies about 3D dose distribution and fluence map prediction were previously done by several groups, and they were treated as two separate problems using different inputs and network architectures [19–29, 32, 33]. Considering dose distribution and fluence maps are highly related, this study took the two tasks in one network. With a minimum extension of 3D U-Net typically used for previous dose distribution prediction task, fluence maps can also be simultaneously generated. The same inputs (3D contours and CT images) for dose distribution prediction were also used for fluence map prediction, no additional inputs such as field doses or pre-calculated 2D feature maps were required.

Currently deliverable plan generation based on solely fluence map prediction is still challenging. Any deviation of fluence prediction would finally compromise the plan quality. Without knowing the achievable patient-specific dose goals, it is difficult to judge whether or not the predicted fluence relates to an optimal plan or the predicted fluence is realistic enough to deliver without significant loss of plan quality. As shown in Fig. 6; Table 1 in this study and several previous studies [24, 27, 29], the plan generated by predicted fluence showed a relatively low target dose coverage and an increased target dose hot spot compared to the ground truth. For these situations, a further plan improvement step would be needed. Thus, the presented study can provide a supplementary solution, the simultaneously generated dose distribution can be either used to guide the further plan optimization. Compared with existing literatures about dose distribution predictions [20, 33], the presented framework achieved similar dose prediction performance and predicted fluence successfully with only one network.

Although the proposed method showed promising results in this study, further validations about the robustness and accuracy of the model are still required. Currently, the model was trained and tested with a dataset including plans with only uniform nine beams, the model performance on other beam configurations for nasopharyngeal carcinoma IMRT need to be validated. Furthermore, the Eclipse Anisotropic Analytic algorithm was used for dose calculation, yet the doses are usually calculated with other dose engines, such as Monte Carlo or Eclipse Acuros XB. Therefore, the model needs to be validated for plans which used a different dose calculation algorithm. In addition, we are also interested in integrating the proposed dose distribution and fluence prediction method in an automatic plan generation scheme for nasopharyngeal carcinoma IMRT treatment.

## Conclusions

We proposed a shared encoder network to predict 3D dose distribution and fluence maps simultaneously for nasopharyngeal carcinoma IMRT patients. The proposed method can be potentially integrated in a fast automatic plan generation scheme by using predicted dose as dose objectives and predicted fluence as a warm start.

## Data Availability

The datasets used during the current study are available from the corresponding author on reasonable request.

## Abbreviations

IMRT: Intensity-modulated radiation therapy
MLC: Multi-leaf collimator
TPS: Treatment planning system
KBP: Knowledge-based planning
DVH: Dose volume histogram
CNN: Convolutional neural network
PTV: Planning target volume
OAR: Organ at risk
MAE: Mean absolute error
SSIM: Structural similarity index
